# Local cone beam CT: how did it all start?

**DOI:** 10.1259/dmfr.20210276

**Published:** 2021-11-09

**Authors:** Yoshinori Arai

**Affiliations:** 1 Department of Oral and Maxillofacial Radiology, Nihon University School of Dentistry 1-8-13 Surugadai Kand Chiyoda-ku, Tokyo, Japan

**Keywords:** Radiology History, Dentistry History, Cone Beam CT, Dental Radiology, Digital Imaging

## Abstract

The mathematical theory of CT was proposed by J. Radon in 1917. It was declared that the projection of whole data sets was needed to reconstruct CT images. Therefore, according to J. Radon’s original theory, local cone beam CT (local CBCT) was impossible to achieve.

In this paper, I discuss how local CBCT was discovered and developed. Its development required many technical elements, such as a turntable and X-ray television system, for basic experiments such as those on which narrow collimation theory and multifunctional panoramic tomography were based. These experiments endured many failures during development.

Now, local CBCT is extremely popular in dental practice because local CBCT has a low radiation dose and high resolution. This paper introduces the technical elements and outlines the important stages during the development of local CBCT in the 1990s.

## Introduction

Local cone beam computed tomography (local CBCT) is extremely popular in dental practice because it administers a low radiation dose and has a high resolution. However, in the past, it was thought to be impossible to develop. This paper introduces the technical elements and outlines the important stages during the development of local CBCT in the 1990s.

## Background prior to local CBCT

**Figure 1.**
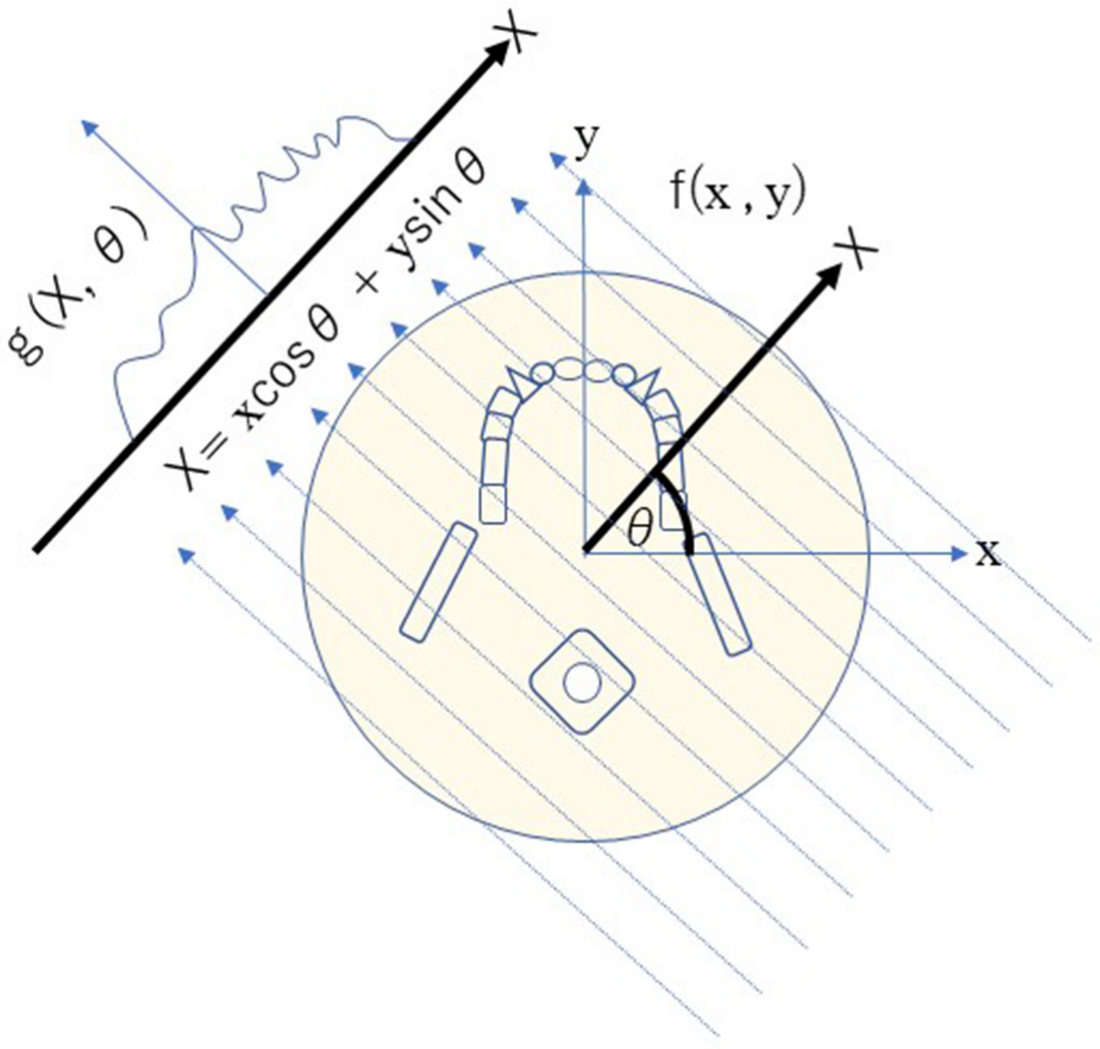
The mathematical theory of CT images are reconstructed from whole projection images from all directions.

In 1917, the mathematical theory of CT was presented by Radon.^1^ He proved that a complete tomographic image can be reconstructed from an infinite set of whole projection images. To obtain the density accurately at a certain point, projection images from all directions and a measurement region ranging from negative infinity to positive infinity are required (Figure 1).

**Figure 2.**
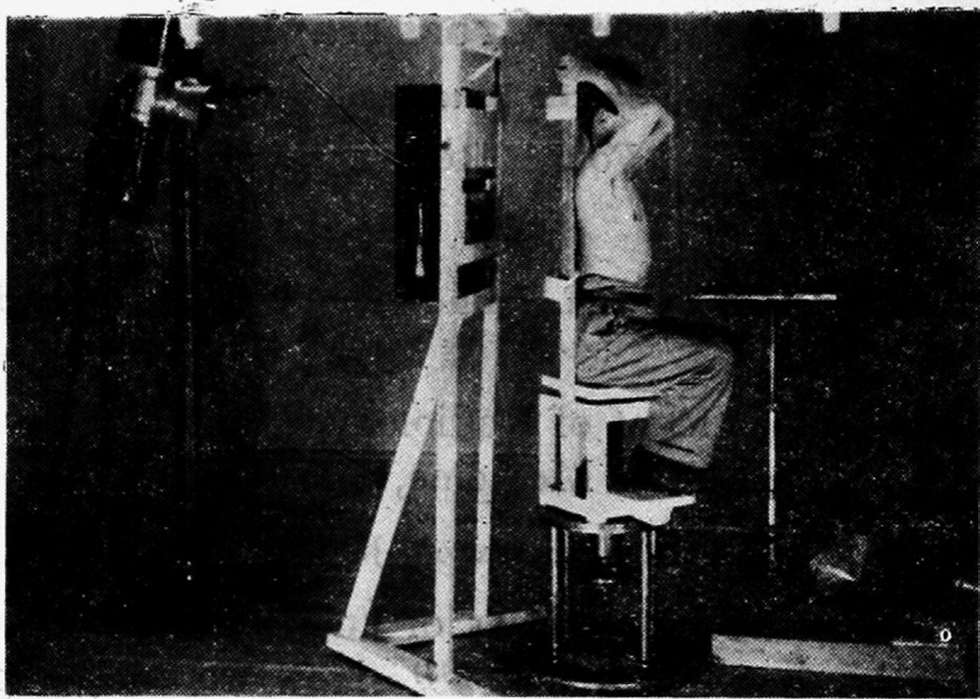
Rotational cross-section tomography. The film is placed on the turntable, and then the X-ray beam is used. *Note*. Reprinted from “Rotatory Crossgraphy”, by Takahashi S. et al, *The Tohoku J. of Experimental Medicine* 1951; 54:60, Figure 1

Takahashi et al developed rotational cross-sectional tomography in the 1950s and used it to obtain cross-sectional images of the chest.^2^ That system employed a turntable on which axial tomography images were acquired on film. The slit was aligned parallel to the plane of rotation, and an X-ray fan beam similar to CT was used (Figure 2). However, that system did not produce sharp images because the tomographic images were blurred.

**Figure 3.**
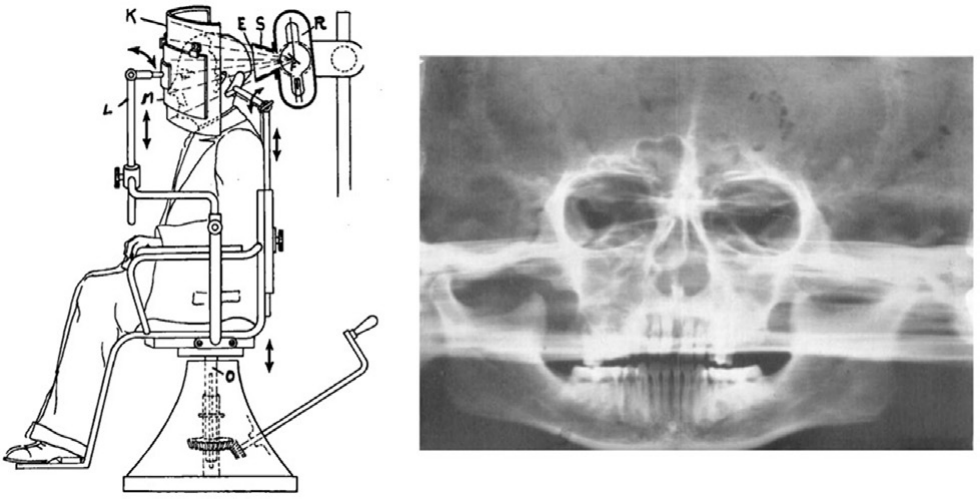
First image of rotational panoramic tomography Note. Reprinted from “A new tomographical method for radiographing curved surfaces”, by Paatero YV.,1949, Acta Radiol 1949; 32:180, Figure 2.

During that time, Paatero developed panoramic tomography, in which the film is set vertically on the turntable. In 1949, he developed the method, successfully acquiring panoramic tomography images of the dental arch and skull.(Figure 3)^3^ This method was similar to Takahashi’s rotational cross-section tomography, which employs a rotating table and slits.

In rotational cross-section tomography, the film and X-ray fan beam are aligned parallel to the plane of rotation. In contrast, in panoramic tomography, the film is aligned perpendicular to the plane of the turntable, the curved surface of film is used, and the slit X-ray beam was aligned parallel to the axis of rotation. These two methods employed similar turntables, but the relationship between the film and the X-ray fan beam was transposed by 90°.

CT was developed by Hounsfield in 1972 in the UK.^4^ Prior to this, it was not possible to image the internal pathology of the brain with X-rays. The brain is surrounded by the skull, which has high X-ray absorption, and therefore, slight differences in X-ray absorption rates between the cerebrospinal fluid, grey matter, and white matter of the brain could not be detected using standard X-ray acquisition methods. CT scans the whole head volume with an X-ray beam and reconstructs the axial images according to Radon’s principle.^1^ Initially, a pencil beam X-ray was used because the sensor had only one channel. Later, the sensor became multichannel, and X-ray fan beams began to be used.

**Figure 4.**
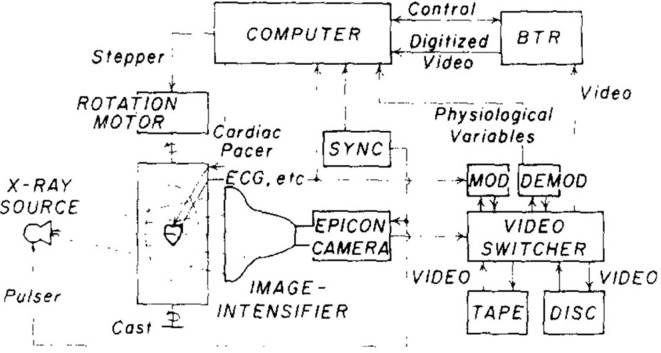
Cone beam CT block diagram by Robb. *Note*. Reprinted from “Three-Dimensional visualization of the intact thorax and contents: A technique for Cross-Section reconstruction from multiplanar X-ray views”, by Robb RA et al., *Comput Biomed Res* 1974; 7:405, Figure 4.

**Figure 5.**
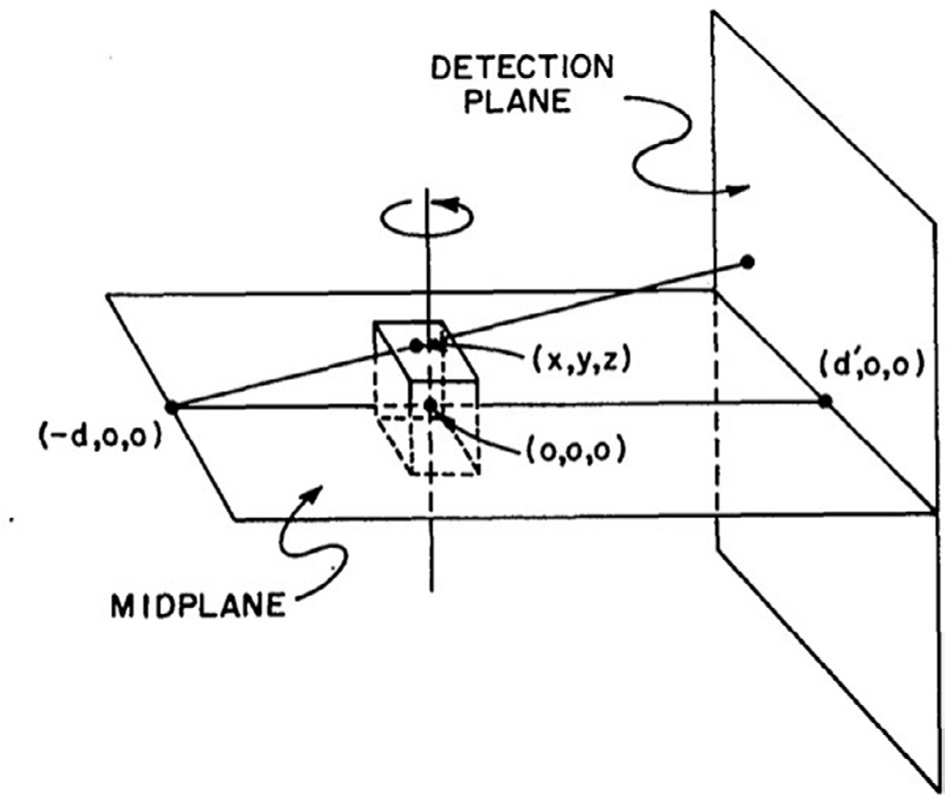
Mathematical theory of cone beam CT by Feldkamp.^6^ *Note*. Reprinted from “Practical cone-beam algorithm”, by Feldkamp LA, et al., *J. Opt. Soc. Am* 1984; 6:613.

**Figure 6.**
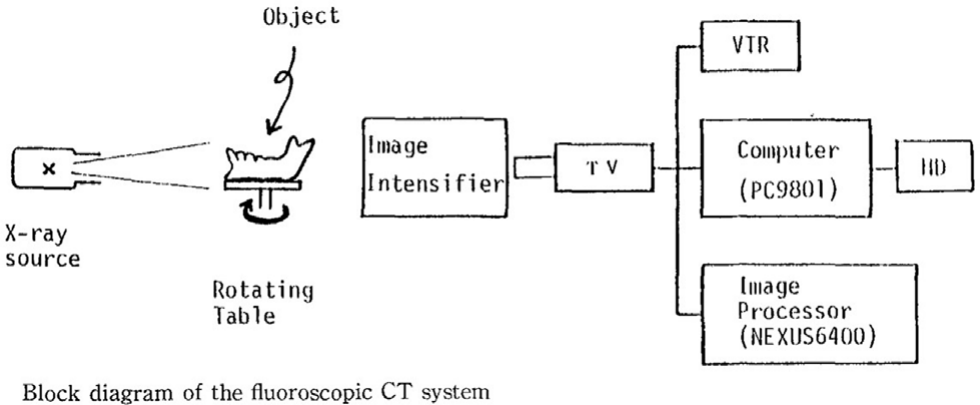
Cone beam CT of jawbone by Toyofuku.^7^ *Note*. Reprined from “Fluoroscopic computed tomography: An attempt at 3-D imaging of teeth and jaw bones”, by Toyofuku F, et al., *Oral Radil* 1986; 2:11, Figure 1.

**Figure 7.**
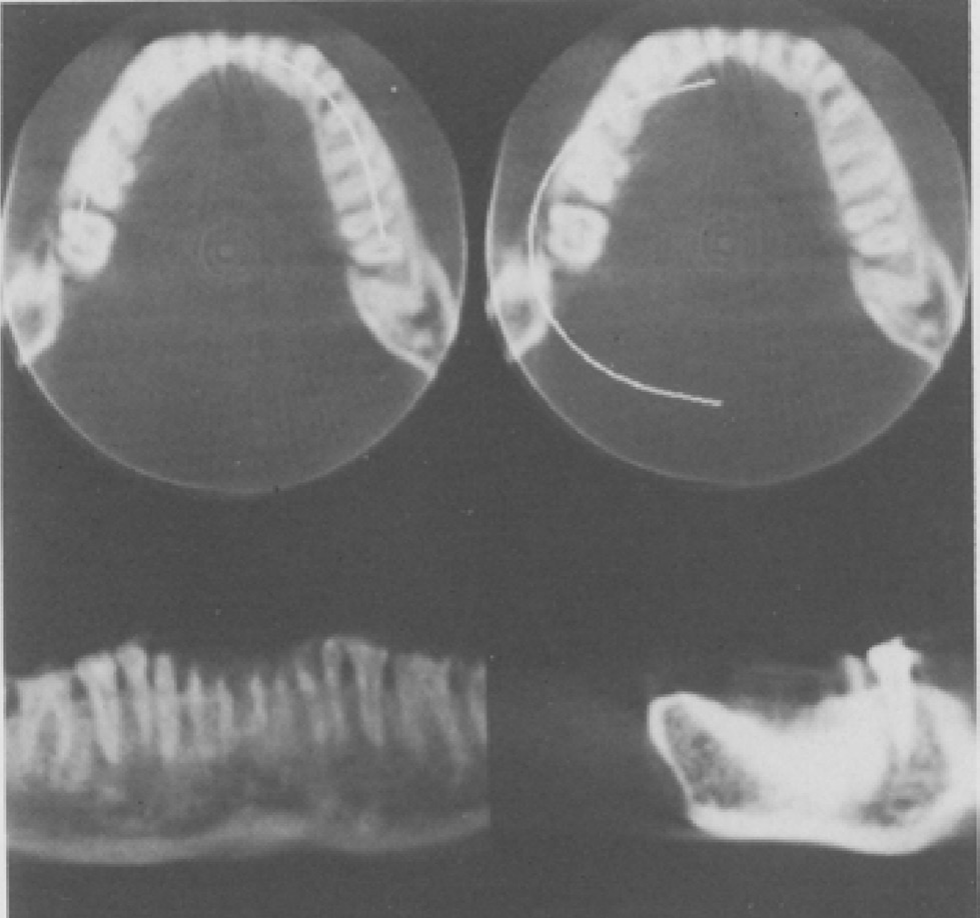
First cone beam CT images of jawbone.^7^ *Note*. Reprinted from “Fluoroscopic computed tomography: An attempt at 3-D imaging of teeth and jaw bones”, by Toyofuku F, et al., *Oral Radil* 1986; 2:12.

**Figure 8.**
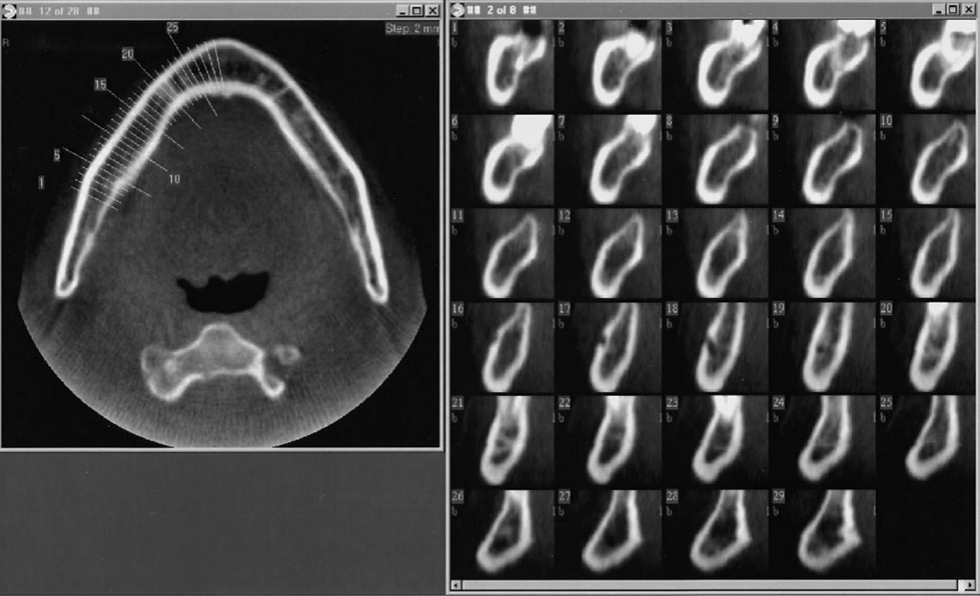
First cone beam CT for clinical application in dentistry. *Note*. Reprinted from “A new volumetric CT machine for dental imaging based on the cone-beam technique: preliminary results”, by Mozzo P, et al., *Eur. Radiol* 1998; 8:1560, Figure 4.

The basic CBCT experiment was performed by Robb et al in 1974 to image the chest and heart (Figure 4).^5^ The basic mathematical algorithm was reported by Feldkamp et al in 1984 (Figure 5).^6^ Toyofuku et al conducted basic research on the application of CBCT to the jawbone using a video television (TV) system and a two-dimensional X-ray Imaging Intensifier (Figures 6 and 7).^7^ Next, a volunteer was placed on the turntable, and axial tomography of the jawbone was successfully performed. This proved that the cone beam system was effective in dentistry. Mozzo et al of Italy developed CBCT as a practical method in 1998.^8^ That system positioned the patient in the bed in supine position and employed an Imaging Intensifier (Figure 8). It was highly effective for pre-operative examination of implants and diagnosis of fractures, and the apparatus was smaller and administered a lower exposure dose than conventional medical CT.

## The local CBCT approach

**Figure 9.**
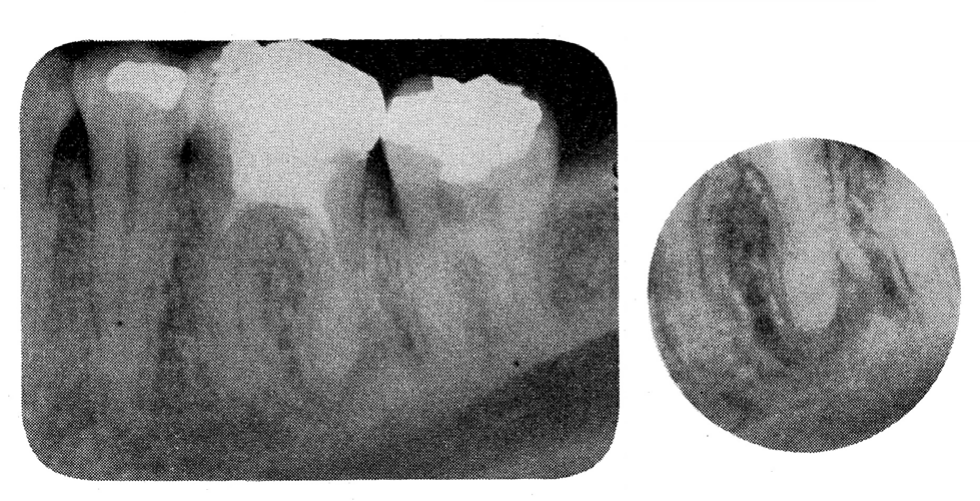
Effects of narrow collimation in intraoral radiography. *Note*. Reprinted from “Effect of narrow collimation on the image representability of periapical bone defects”, by Nishiyama S., *Dental Radiology* 1977; 17:27, Figure 10.

The exposure field of typical intraoral radiography has a diameter of approximately 6 cm, but Nishiyama reported that the image sharpened when the diameter of the exposure field was shrunk to 2 cm.^9^ In particular, the minute structure of the apex was captured in detail (Figure 9). Ando et al developed an intraoral X-ray system that employed a high-sensitivity TV system, a Silicon Intensifier Target Tube, and an X-ray head in a narrow collimation exposure field.^10^ This method also adopted the field with diameter of 2 cm, and the resulting images could be observed immediately with exceptionally low radiation exposure (Figure 11). This was one of the main root innovations that paved the way for local CBCT.

**Figure 10.**
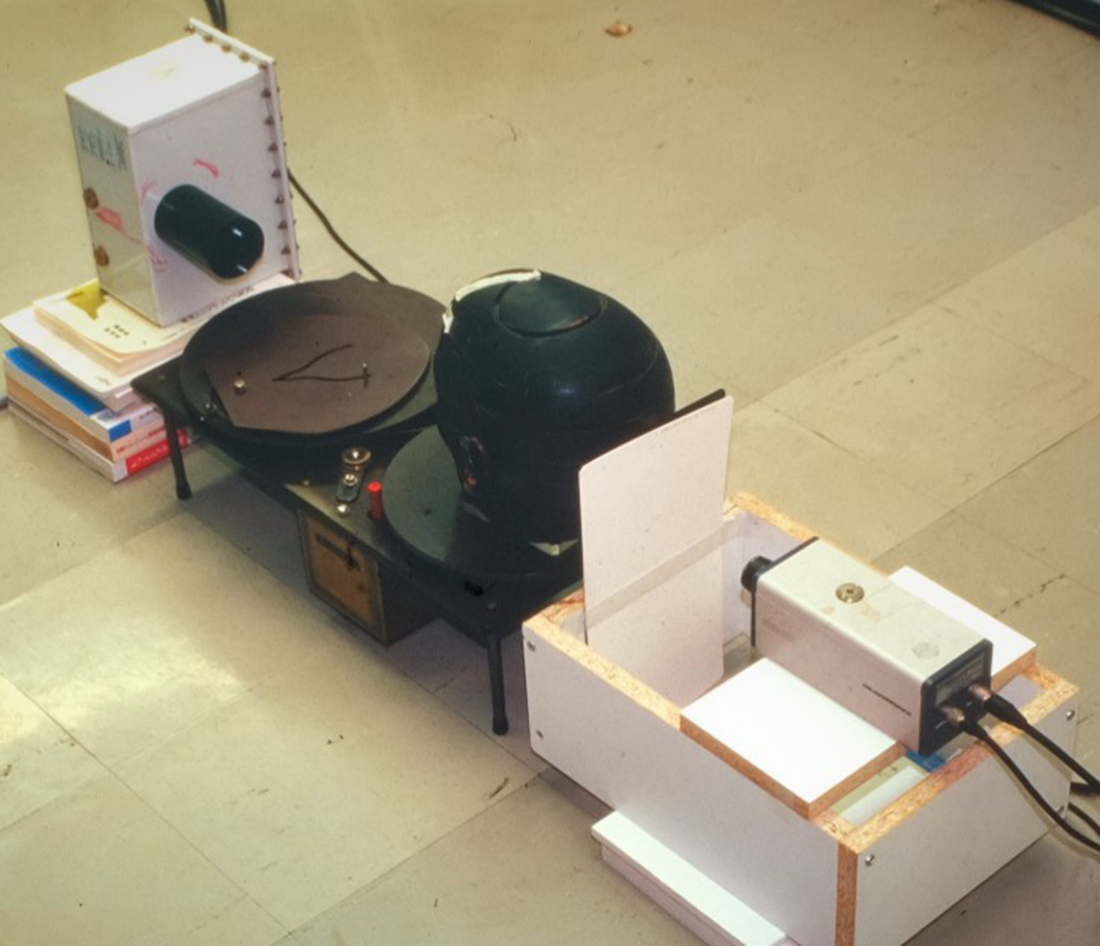
First local cone beam CT experiment Paatero’s turntable was used in this experiment. *Note*. Reprinted from “100 years + a century of innovation”, by Morita H., Tokyo, Japan: Toppan publisher; 2020. pp. 381.

*Zonarc*^®^ (Palomex Oy, Helsinki, Finland) was developed as a multifunction panoramic tomography method in the 1980s.^11^ Similar to medical CT, an X-ray tube revolved around the head. The slit X-ray beam was exposed parallel to the axis of rotation.

**Figure 12.**
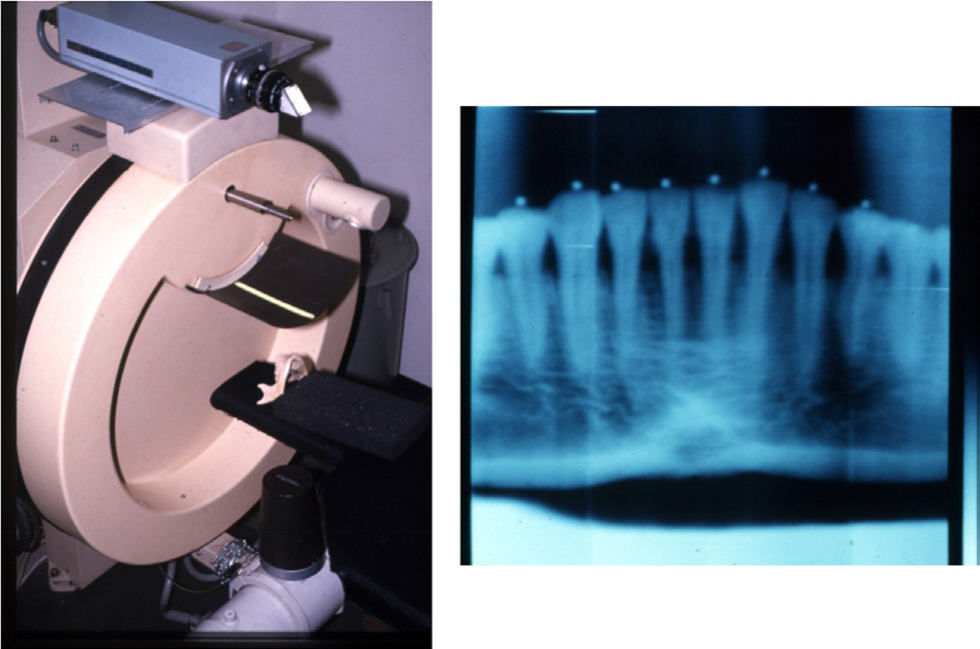
Basic research on digital panoramic tomography. *Note*. Reprinted from “An improvement digital panoramic tomography system” by Arai Y., *Dental Radiology* 1988; 28:304, Figure 2.

**Figure 13.**
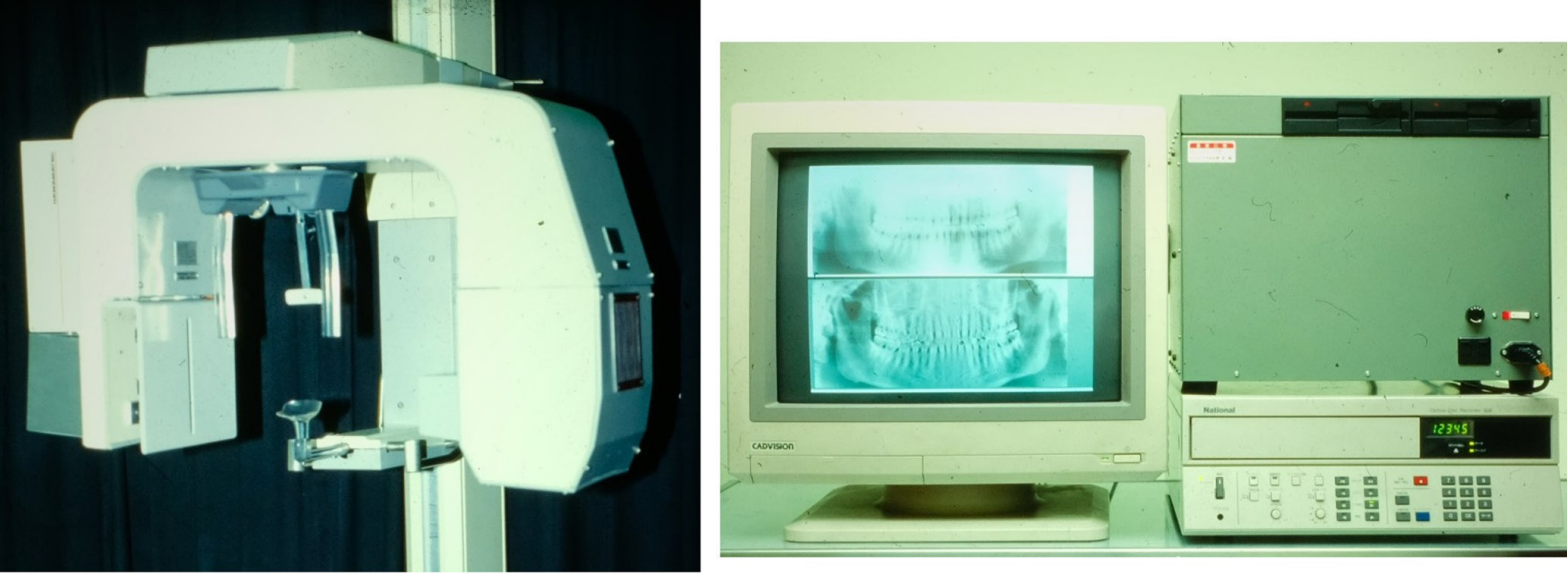
Prototype of digital panoramic tomography. This system failed because of excessive radiation doses and poor image quality. *Note*. Reprinted from “The elimination of ghost images in the development of digital rotational panoramic radiography”, by Arai Y., *Dental Radiology* 1994; 34:196, Figures 1 and 2.

Arai remodeled the *Zonarc*^®^ system and developed digital panoramic tomography. The latter method was used on the film to implement a high-sensitivity TV system. The slit image was converted to a digital signal at 30 frames per second and saved as frame images. The data were shifted and added to perform the panoramic tomography reconstruction. By changing the amount of shift, a panoramic tomographic layer of any surface can be obtained (Figure 12).^12^ Then, in 1992, a prototype digital panoramic system was developed (Figure 13).^13^ However, the limited technology available at that time resulted in low sensor sensitivity, and thus the noise level was high, and the computer’s performance was slow. Therefore, the exposure dose was large, and the image quality was poor compared with that of conventional film-based panoramic tomography. For practical purposes, digital panoramas could not be used simultaneously with this technology. However, this experience was especially useful for the later development of local CBCT.

In the 1980s, Tammisalo et al developed a multifunctional scanner named the *Scanora^®^* (Soredex Oy, Helsinki Finland).^14^ It was an epoch-making device that enabled tomography by circular, pendulum-like motion of the C-shaped arm that connected the ^®^ tube to the sensor. The *Scanora*^®^ was the prototype for local CBCT.^15^ In 1994, J. Morita Mfg. Co. developed a multifunctional tomography system called the *veraviewScope^®^*. This device had a mechanism to move the rotational axis of the rotation arm in the XY plane. As a result, linear tomographic images could be obtained at arbitrary positions on the dental arch. However, the films contained stacks of blurred images, and thus, the image quality was not sufficient for diagnosis. This triggered the development of local CBCT for dental use.

In the latter half of the 1980s, third-generation CT came into use in dental hospitals.^16^ However, it did not have sufficient resolution compared with intraoral radiography. Additionally, as the CT voxels were rectangular parallelepipeds, the partial volume effect was large. In certain cases, the resolution of the coronal and sagittal sections was low, and the resolution was insufficient for diagnosis in evaluation for procedures such as root canal treatments. Furthermore, the exposure dose was several hundred times higher than that of intraoral radiography. These were some of the many major problems in using that generation of CT techniques in dental practice.

The development of CT for dental use, such as imaging the temporomandibular joint (TMJ) and endodontics, has therefore been desired. High resolution and low exposure were required for clinical application in dentistry, in addition to low cost. Therefore, it was considered to be technically impossible to develop.

### Development of local CBCT

Arai started developing local CBCT around 1993 after the development of the digital panoramic system failed.^13^ The triggering event was that Dr K. Honda requested that I provide three-dimensional visualization of the TMJ. However, the research could not progress until I studied abroad at University of Turku, Finland in 1995. This University was noted for the invention of panoramic tomography by Paatero.^3^ The basic local CBCT experiment employed a turntable, which was the principle of the panoramic tomography research by Paatero in the 1940s (Figure 10).^17,18^

**Figure 11.**
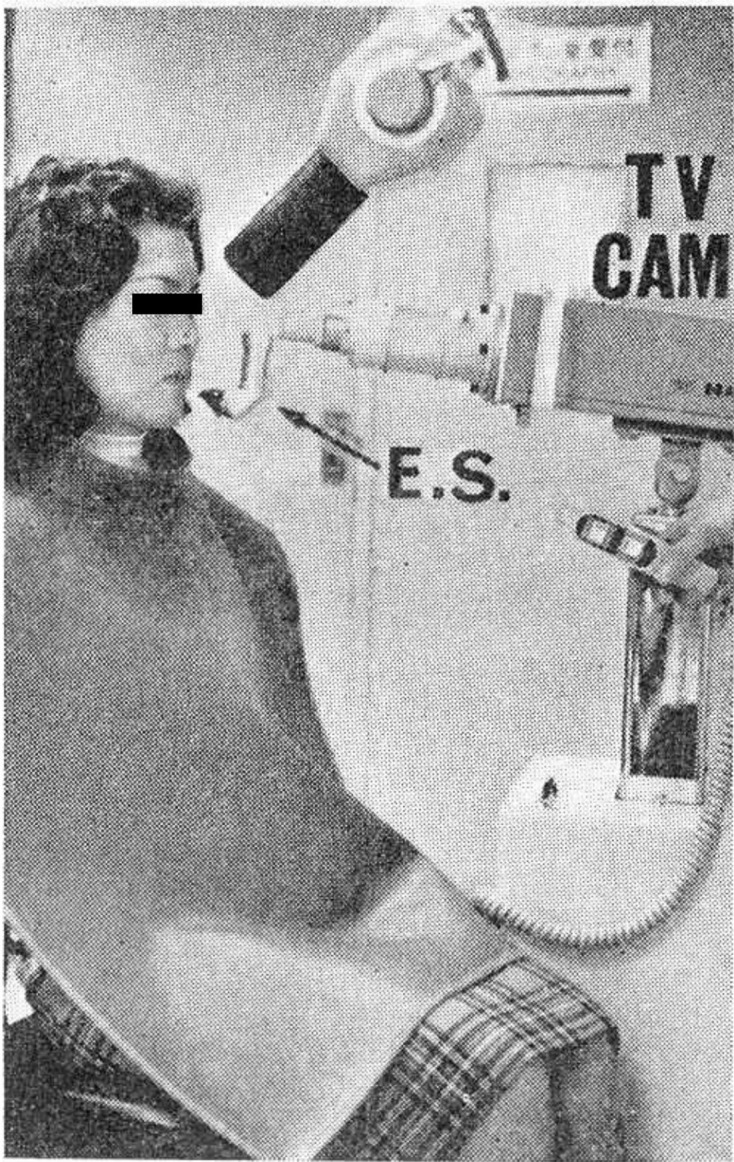
Intraoral X-ray TV system. *Note*. Reprinted from “Real Time radiological survey by intraoral fluoroscopic TV system to minimize radiation dose”, by Ando S, et al., *Dental Radiology* 1978; 18:125, Figure 2.

**Figure 14.**
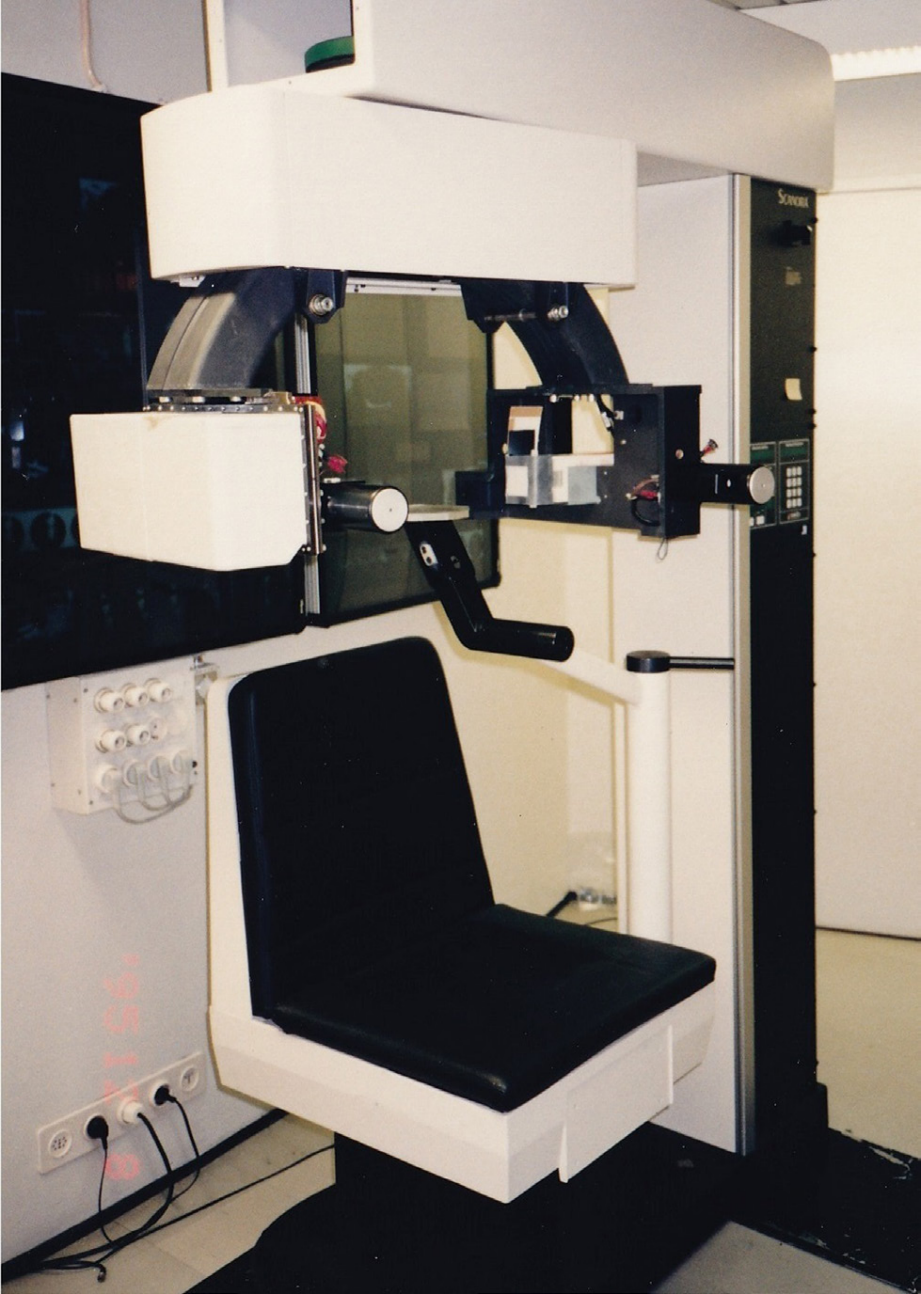
Use of *Scanora^®^* in Local cone beam CT research.

**Figure 15.**
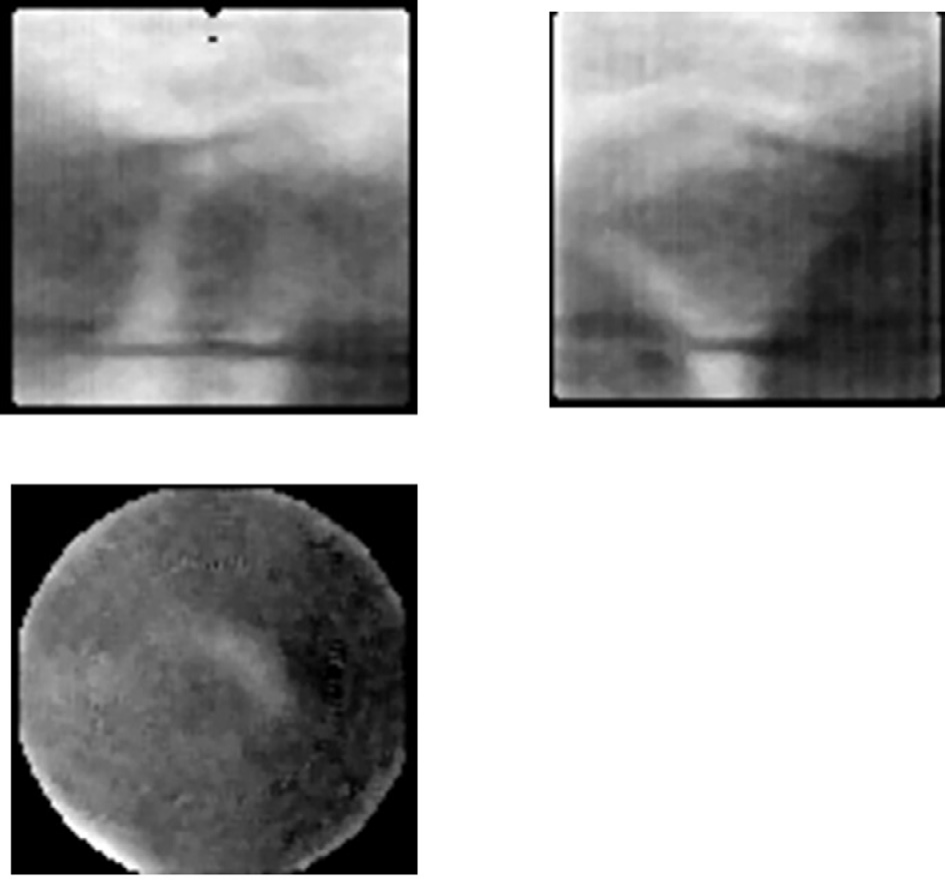
First local cone beam CT images Images of the TMJ of a *Rando^®^* phantom were acquired. The image quality was poor. However, this experience proved that local CBCT could reconstruct some images.

After that, the basic *Scanora*^®^ experiment was conducted by incorporating a high-sensitivity TV system, which employed the failed digital panoramic system from 1992^13^ (Figure 14). The object was the TMJ of a *Rando^®^* phantom (Alderson, CT). First, images of the TMJ were acquired,(Figure 15) but the image quality was extremely poor (^®^). However, this experiment showed that it was possible to reconstruct a three-dimensional tomographic image even with a local of projection images.

**Figure 16.**
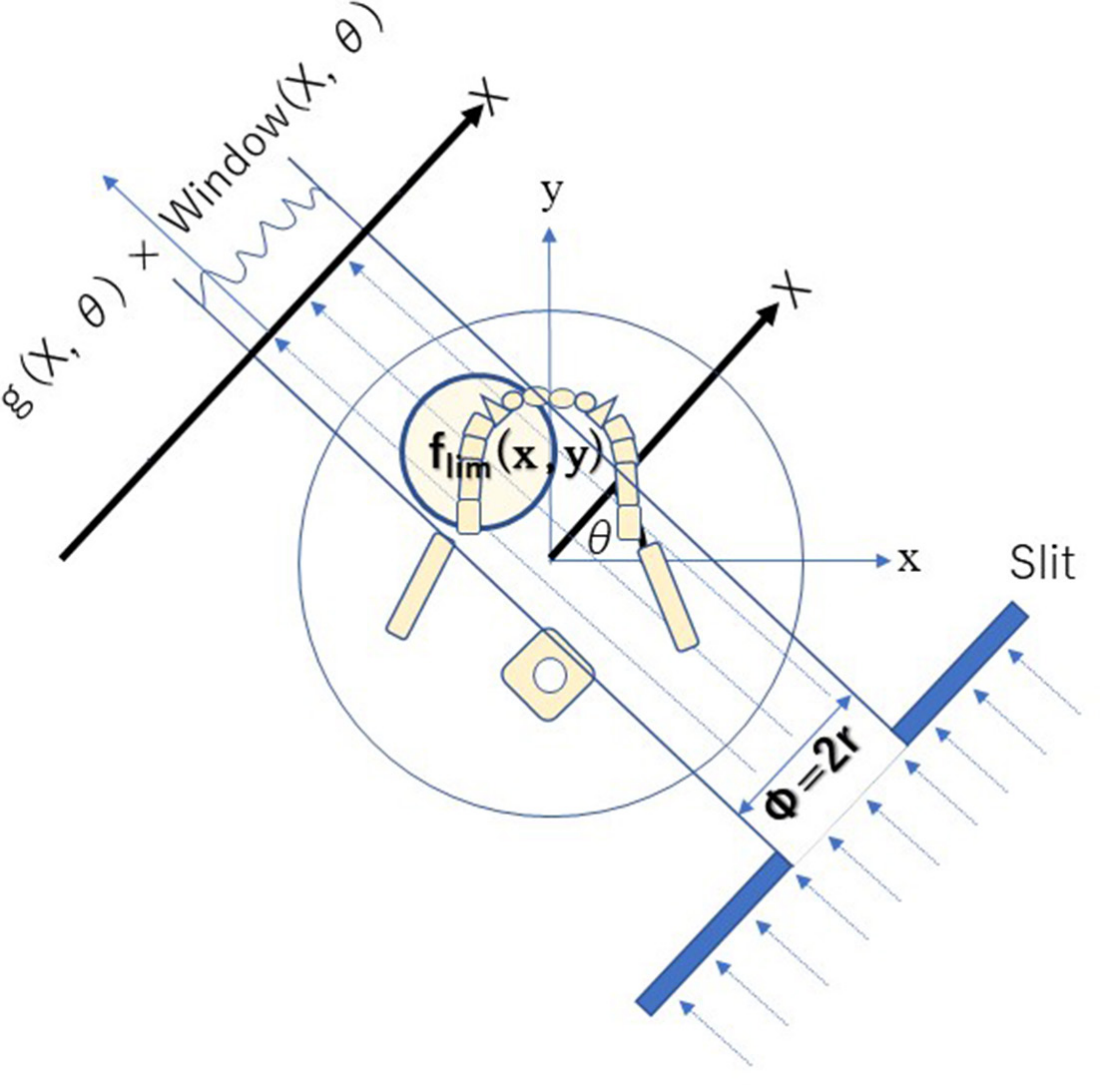
Limited projection images from local cone beam computed tomography If the slit X-ray beam is blocked, then local CBCT cannot detect whole projection images.

According to Radon’s theory,^1^ a local projection images of the whole head cannot be obtained (Figure 16). Therefore, tomographic images were thought to be impossible to reconstruct based on such local projections. However, this experiment showed that such images could be reconstructed under special conditions.

To image soft tissues such as muscle and fat, it is necessary to accurately detect slight differences in the X-ray absorption rate. However, bones and teeth have a high X-ray absorption rate, and therefore their imaging has higher contrast than that of soft tissue. Thus, even if the X-ray absorptivity rate cannot be accurately obtained, an image can still be visualized if the relative absorptivity difference is obtained with some error.

In local CBCT, because of the above considerations, images of low-contrast soft tissue are not produced from the local projection images, but images of high-contrast hard tissue are. Nevertheless, an accurate X-ray absorption rate is not calculated, indicating that accurate CT values cannot be obtained in local CBCT.

**Figure 17.**
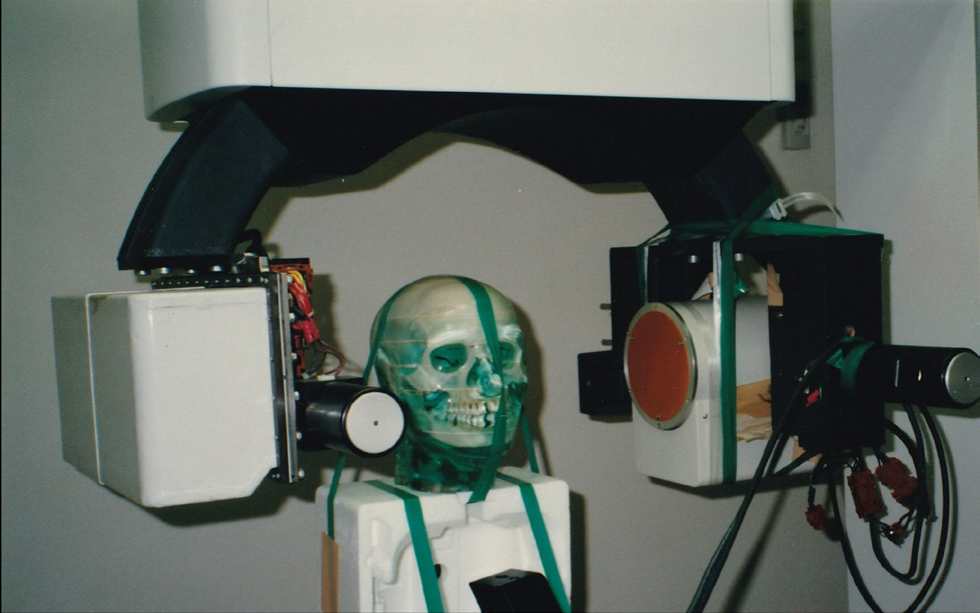
Prototype of local cone beam CT. An image intensifier with a diameter of 10 cm was mounted on the *Scanora^®^*.

**Figure 18.**
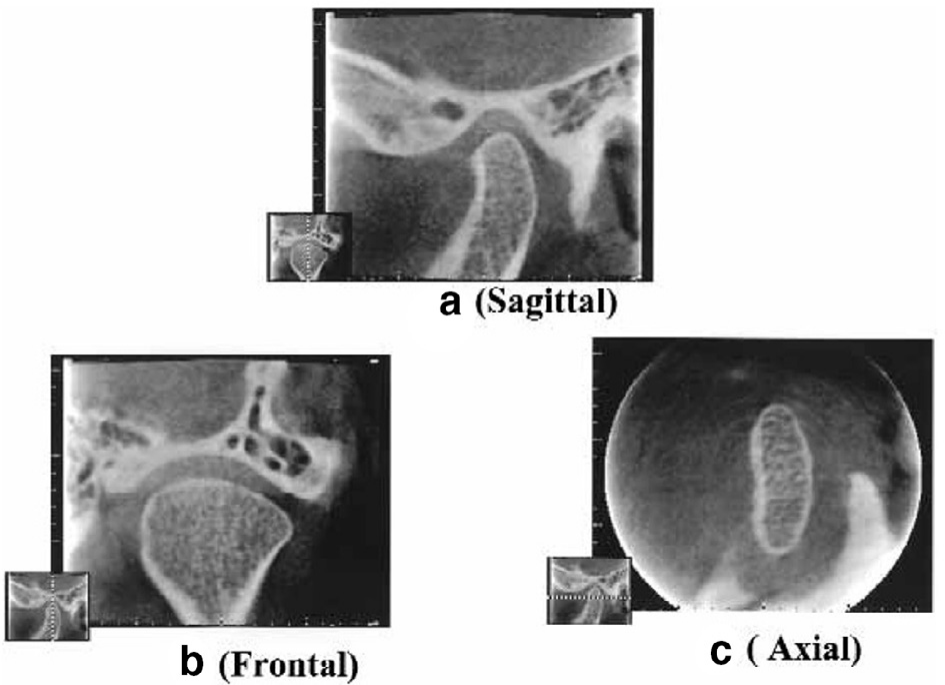
TMJ images created using the *Ortho-CT* prototype, Note. Reprinted from “Ortho cubic super-high resolution computed tomography: A new radiographic technique with application to the temporomandibular joint”, by Honda K. et al., *Oral Surg Oral Med Oral Pathol Oral Radiol Endod* 2001; 91:240, Figure 2. TMJ, temporomandibular joint.

Next, an Image Intensifier with a diameter of 10 cm was integrated into the *Scanora*^®^, which provided further improvement (Figure 17). The reconstruction software was written in the C language, but floating-point mathematics was not used to speed up the calculations. Instead, dedicated binary fixed-point arithmetic was programmed by Arai. Finally, with the release of the Pentium^®^ II 400 MHz (Intel Co., CA) CPU in 1997, it became possible to reduce the reconstruction time to less than 10 min. In December 1997, clinical research began at the Department of Oral and Maxillofacial Radiology, Nihon University of School of Dentistry Dental Hospital. The nickname of the local CBCT prototype was Ortho-CT because the voxel shape was ortho cubic. Honda et al reported that the method made it possible to acquire three-dimensional images of the TMJ (Figure 18).^19^ At that time, the goal was to conduct local CBCT in 1000 cases annually because the price of the local CBCT device was predicted to be approximately 500,000 US dollars. If such a device is amortized over 5 years, the cost of 1 examination is 100 US dollars if 1000 cases are conducted annually. To put this method into practical use, it was necessary to consider the associated medical costs. Fortunately, 2500 cases were conducted in the first 3 years, and an annual rate of more than 1000 cases could be accommodated 2 years later. According to the literature, the goal was achieved.^19,20^

The radiation dose of local CBCT was measured using an Alderson *Rando^®^* phantom. When a local exposure with a diameter of ^®^ cm and a height of 3 cm was used, its dose was several times that of panoramic tomography but only approximately 1/100 of that of conventional CT.^20^

The spatial resolution of local CBCT was calculated via the modulation transfer function using the wire method. It was as good as 10% at 2.0 line pare/mm.^20^

**Figure 19.**
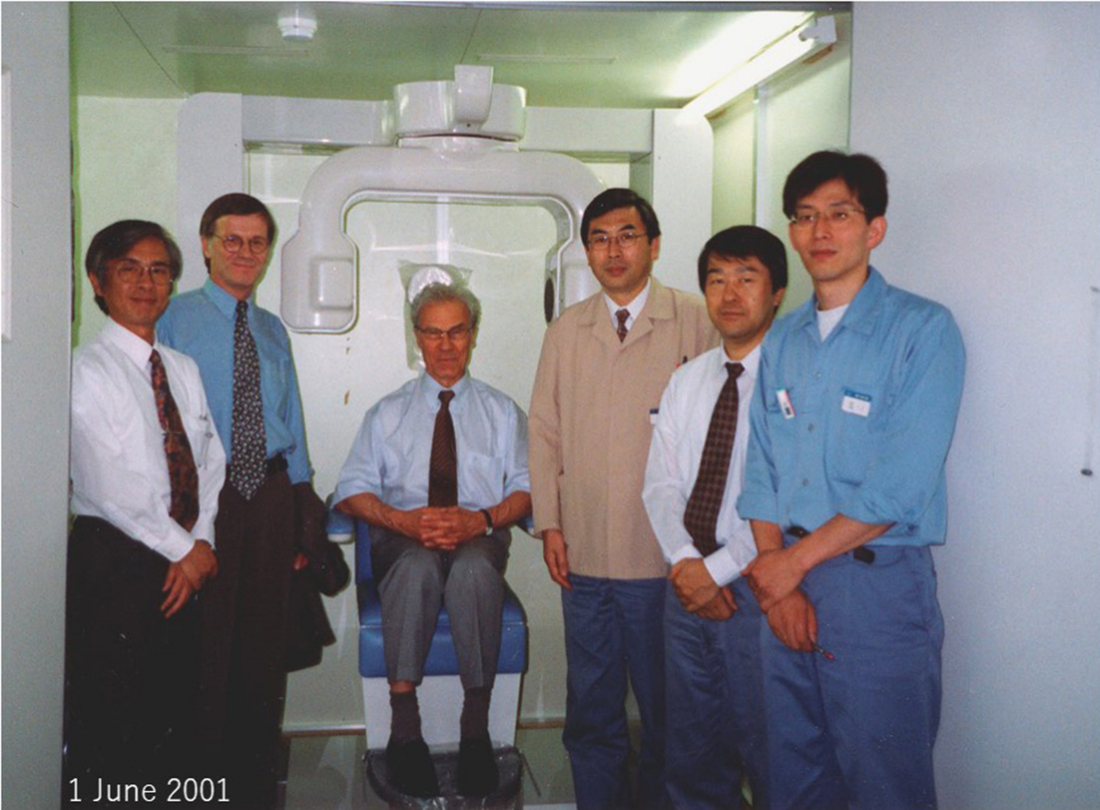
Completed local cone beam CT Center: Professor E. Tammisalo named the system “*Accuitomo*” alongside the development team of J. Morita Mfg. Co. on June 1, 2001.

The license for this technology was transferred to J. Morita Mfg. Co. from the Nihon University Business Research and Intellectual Property Center. Local CBCT was developed as a commercial device, and its safety and efficacy were indicated by the above results. Thus, regulatory approval was obtained from the Ministry of Health, Labour and Welfare (Japan) in December 2000. It was named “*Accuitomo^®^*,” which means ^®^accuracy and tomography,” by Professor E. Tammisalo of the University of Turku (Figure 19).

## Conclusion

At the time of development, I thought that “CT using local projection images,” which could only obtain incomplete projection data, could not be put to practical use because of the occurrence of artifacts. However, it is now widely applied in dentistry as a method that achieves low exposure and high resolution. In particular, it is often used for endodontic treatment and diagnosis of the TMJ.^19,21,22^

Without the suggestion from Honda,^19^ the turntable employed by Paatero,^3,17^ the *Scanora^®^* system by E. Tammisalo,^14^ the ^®^ TV system by Ando,^10^ narrow collimation theory by Nishiyama,^9^ and the failure of the development of digital panoramic tomography by myself^13^ and many pioneering researchers, local CBCT would not have succeeded. Therefore, I am very grateful to all of them.
